# Bridging Pedagogy and Technology: A Systematic Literature Review on Inquiry-Collaborative Models Assisted with Virtual Laboratory in Physics Education

**DOI:** 10.12688/f1000research.191181.1

**Published:** 2026-10-07

**Authors:** Fahmi Yahya, Rusijono Rusijono, Utari Dewi, Kartika Metafisika, Winda Lestiani, Hermansyah Hermansyah, Dens E. S. I Asbanu

**Affiliations:** 1 Department of Educational Technology, Universitas Negeri Surabaya, Surabaya, East Java, 60231, Indonesia; 2 Science Education Study Program, Universitas Pendidikan Indonesia, Bandung, West Java, 40154, Indonesia

**Keywords:** virtual laboratory, inquiry-based learning, collaborative learning, physics education, systematic review, instructional design, evidence synthesis.

## Abstract

**Background:**

Virtual laboratories have become a standard infrastructure in physics teaching, yet their pedagogical value depends on the learning model that frames them. Inquiry and collaborative learning are routinely invoked together, but no synthesis has treated them as the joint pedagogical framework for virtual laboratories in physics education or examined how the three are configured in practice.

**Methods:**

Scopus was searched on July 9, 2026, for English-language journal articles published between 2017 and 2026, reporting primary studies in formal physics education that used a virtual laboratory, interactive simulation, or digital experimental environment within an inquiry and/or collaborative framework and reported at least one learning outcome. The Reporting follows Preferred Reporting Items for Systematic Reviews and Meta-Analyses (PRISMA) 2020. Quality was appraised from full texts using the Mixed Methods Appraisal Tool version 2018, and the findings were synthesized through a six-phase thematic analysis.

**Results:**

In total, 925 records were returned. Of the 110 reports sought, 23 were not retrieved and 87 were assessed in full text, giving 58 studies in 59 reports from 22 countries, most often in higher education (n = 27) and upper secondary school (n = 22), with quasi-experimental designs being dominant (n = 21). Combining real and virtual laboratories consistently outperformed either single mode, while virtual laboratories alone matched physical laboratories on conceptual mastery and surpassed them on abstract concepts and inquiry self-efficacy. Guidance, moderated by prior knowledge, determines whether exploratory freedom aids learning or adds cognitive load. Conceptual outcomes were measured in 45 studies (76.3%), but collaboration was designed explicitly in only nine reports (15.3%) and measured in five (8.5%).

**Conclusions:**

The advantage of virtual laboratories comes from the quality of the pedagogical design around them, and not from technology. Collaboration is the component least often designed and measured, so the evidence base remains one about inquiry with virtual laboratories that happen to be delivered in groups.

## 1. Introduction

Laboratory work is the core of learning physics. Through these activities, learners not only prove existing theories, but also practice formulating questions, controlling variables, analyzing data, and building arguments from evidence ( Hofstein & Lunetta, 2004). Owing to the limited physical facilities in many educational institutions and the rapid growth of online learning, this role is gradually shifting to virtual laboratories. In these digital simulation environments, students can manipulate variables and observe results without touching physical equipment ( de Jong et al., 2013). Critical reviews report that digital simulations can match or exceed conventional instruction on conceptual mastery, where the simulation is within adequate instructional design ( Rutten et al., 2012; Smetana & Bell, 2012). Similarly, Banda and Nzabahimana (2021) emphasize that the improvement in understanding from using simulations such as Physics Education Technology (PhET) depends heavily on how the tools are integrated into the classroom, not simply on their availability. Technology is simply a potential enabler; this potential only becomes a meaningful learning experience when it is driven by the right teaching model.

Inquiry-based learning is the model that is most often paired with virtual laboratories. Pedaste et al. (2015) synthesized its variants into a five-phase cycle of orientation, conceptualization, investigation, conclusion, and discussion. Furtak et al. (2012) reported that an inquiry approach yielded better results than conventional teaching, with the greatest benefits achieved when there was direct guidance from the teacher. The degree of guidance was also tested. Kirschner et al. (2006) argued that insufficient guidance can place an excessive cognitive burden on beginner students. Hmelo-Silver et al. (2007) replied that an inquiry approach accompanied by structured support (good scaffolding) can effectively manage this burden. Furthermore, Lazonder and Harmsen (2016) demonstrated that providing guidance is helpful, but its success is largely determined by the appropriateness of the type of guidance provided to students’ characteristics and needs.

Collaboration is the second dimension of laboratory practice. Laboratory work is rarely done individually; it typically takes place in small groups where learners discuss meaning, divide roles, and consolidate their understanding in solving problems ( Roschelle & Teasley, 1995). However, sharing a bench is not the same as collaborating. Dillenbourg (1999) draws the line of whether partners work on the problem together or divide it and proceed in parallel, a distinction that runs through the findings reported below. Social interdependence theory explains why cooperative structures outperform competitive and individualistic ones ( Johnson & Johnson, 2009). In the digital realm, a meta-analysis conducted by Chen et al. (2018) found that the effects of computer-supported collaborative learning depend on the strategies that support it and not on the technology used. Complementing this view, the ICAP framework ( Chi & Wylie, 2014) places interactive activities at their highest level on the student engagement scale.

Although theoretically, these two dimensions complement each other, reviews tend to analyze them separately. Research generally focuses on a single key issue, such as comparing physical and virtual laboratories, evaluating the effectiveness of a particular simulation, or simply discussing inquiries as a learning model. None has treated inquiry *and* collaborative learning as the joint pedagogical framework for virtual laboratory use in physics education or examined how the three are configured in practice. The gap matters for education systems that, like Indonesia, promote learner-centered instruction while facing limited laboratory provision. This review addresses the following four questions: RQ1: What characterizes the studies applying inquiry-collaborative models assisted with virtual laboratories in geographical spread, educational level, research design, and methodological quality? RQ2: How are inquiry and collaborative learning configured alongside virtual laboratory technology? RQ3: Which learning outcomes are reported and in what direction? RQ4: Which moderating factors and implementation constraints can be identified from the evidence?

## 2. Methods

### 2.1 Design and eligibility

This literature review was based on the Preferred Reporting Items for Systematic Reviews and Meta-Analyses (PRISMA) 2020 guidelines ( Page et al., 2021). The implementation of this guideline covers 27 checklist points, the detailed mapping of which can be seen in Appendix B. Before the literature search process began, an internal protocol containing research questions, eligibility criteria, search strategies, and synthesis methods was designed. The literature eligibility criteria were formulated using the PICo framework, and are listed in Table 1. In brief, studies were included if they were English-language journal articles published between 2017 and 2026; reported a primary study in formal physics or physical science education; used a virtual laboratory, interactive simulation, or digital experimental environment including augmented and virtual reality; applied an inquiry and/or collaborative model as the instructional frame (the 5E learning cycle as specified by Bybee et al., 2006); and reported at least one learning outcome for learners.

**Table 1.** Eligibility criteria under the PICo framework.

Parameter	Included	Excluded
**Population (P)**	Physics learners at primary, lower secondary, upper secondary, or higher education level, including prospective teachers	Instructors as the only data source; participants outside formal education
**Interest (I)**	An inquiry model (guided, structured, or scaffolded inquiry, POGIL, the 5E learning cycle, discovery learning) and/or collaborative learning, assisted by a virtual laboratory, interactive simulation, augmented or virtual reality, or remote laboratory	Technology without an instructional model; an instructional model without a digital laboratory component
**Context (Co)**	Formal physics or physical science learning: mechanics, electricity and magnetism, optics, thermodynamics, waves, modern physics	Chemistry, biology, mathematics, engineering, or pure computing; non-formal education and public outreach
**Outcome**	At least one learner outcome from the eight competency categories listed in Subsection 2.4	Product validity, expert perception, or media feasibility only, with no learner outcome data
**Publication**	Scopus-indexed journal articles reporting primary studies, published 2017 to 2026, in English	Conference papers, book chapters, editorials, reviews, meta-analyses, bibliometric studies; published before 2017

PICo = Population, Interest, Context. POGIL = Process-Oriented Guided Inquiry Learning. Conference papers were retrieved by the query and excluded at first screening.

**Table 2.** Item-level MMAT appraisal of the 59 reports, by design category.

MMAT category	Reports	Criteria most often met	Criteria least often met
Quantitative non-randomised	23	Measurements appropriate (20); complete data (18)	Participants representative (2); confounders accounted for (13)
Quantitative descriptive	14	Analysis appropriate (9); sampling relevant (8)	Sample representative (0); nonresponse bias low (2)
Randomised trials	12	Groups comparable at baseline (9); adherence (9)	Assessors blinded (2); randomisation described (4)
Mixed methods	8	Rationale for the design (6)	Each strand meets its tradition (0); divergences addressed (2)
Qualitative	2	Approach appropriate (2); collection adequate (2)	Findings derived from data (1)

MMAT = Mixed Methods Appraisal Tool ( Hong et al., 2018). Bracketed figures are the number of reports meeting that criterion. Each report is judged only against the five criteria of its own design category. The full grid is Table 5.

**Table 3.** Synthesis themes mapped to the research questions, with supporting evidence and certainty.

Theme	RQ	Support	Direction	Certainty
Combined modalities outperform single modalities, which are themselves broadly equivalent	RQ3	21 reports (20 studies)	Consistent. Real-virtual combinations superior; virtual and physical equivalent on conceptual mastery, with domain-specific advantages	High
Graded guidance determines the effectiveness of digital inquiry	RQ4	17 reports	Consistent and aligned with meta-analytic evidence outside the corpus; moderated by prior knowledge	High
Collaboration is widely declared but rarely designed and measured	RQ2, RQ4	9 reports design collaboration; 5 measure a collaborative outcome	Consistent, but too few studies to generalise; best read as a research gap	Low
The competency spectrum extends beyond the cognitive domain	RQ3	59 reports (58 studies)	Positive and consistent in the cognitive domain; divided in the affective domain	Moderate
Contextual preconditions govern implementation	RQ4	14 reports (13 studies)	Resting on specific contexts whose transfer has not been tested	Moderate

Certainty follows four GRADE-adapted considerations: number of supporting studies, consistency of direction, methodological quality, and transferability. Counts overlap across themes because a report may support more than one theme.

**Table 4.** Characteristics of the 58 included studies reported in 59 reports.

ID	Study	Country	Level	Design and sample	Virtual laboratory technology	Learning model	Competencies measured	Main finding
118	Abuna et al. (2026)	Ethiopia	Upper sec.	Three-group quasi-experiment; 150 grade-10 students (IBVLE 45, IBPLE 49, IBNLL 56)	Virtual lab vs physical lab vs non-lab	Inquiry	Cognitive	Inquiry-based virtual lab (IBVLE) produced the largest gains in mechanics understanding
191	Adimayuda et al. (2025)	Indonesia	Higher ed.	Convergent parallel mixed methods; 22 first-semester undergraduates (14 male, 8 female)	PhET + video + AI-assisted reflection tool (T-MORE)	Reflective inquiry	Cognitive	McNemar test significant; high-category reduction of momentum–impulse misconceptions
309	Alabidi et al. (2023)	United Arab Emirates	Upper sec.	Pretest–posttest quasi-experiment; 90 grade-11 students (experimental 45, control 45)	Computer simulations	Inquiry	Cognitive	Large effect sizes on conceptual understanding of Newton's second law (d = 2.10 female; d = 2.94 male)
182	Alstein et al. (2026)	Netherlands	Upper sec.	Lesson study; 42 pre-university students (28 grade 12 at school A; 14 grade 11 at school B)	Special-relativity simulation environment	Simulation-based inquiry	Cognitive	Simulations surfaced and sharpened cognitive conflict; interpreting relativistic behaviour of light remained difficult
164	Auliya et al. (2025)	Indonesia	Upper sec.	R&D (4D, through the Develop stage); 42 students at a state upper-secondary school in Bandar Lampung, 2 teachers, 6 experts	Phyphox-integrated e-worksheet	PBL	Collaborative	Media feasibility 88.7% and content 93.3%; practicality 93.0–95.2%; effectiveness not yet tested
925	Bahtiar et al. (2022)	Indonesia	Lower sec.	One-group pretest–posttest pre-experiment; 30 grade-IX students, MTs Hidayatullah Mataram	PhET (static electricity)	Discovery learning	Cognitive (sci. literacy)	Female students' scientific literacy (80) exceeded that of male students (77.95)
451	Banda and Nzabahimana (2023)	Malawi	Upper sec.	Quasi-experiment; 280 students (control 136, experimental 144)	PhET interactive simulations (oscillations and waves)	Simulation-based learning	Cognitive; Affective	PhET group outperformed on motivation and academic achievement
464	Ben Ouahi et al. (2024)	Morocco	Lower sec.	Three-group quasi-experiment; 166 students aged 13–15 from 6 classes (RL 58, VL 56, RL+VL 52)	Real lab, virtual lab, and their combination	5E learning cycle	Cognitive	The RL+VL combination produced the most substantial gains in electricity learning outcomes
430	Castillo-Salazar et al. (2026)	Spain	Upper sec.	Five-phase didactic design research; Upper-secondary students	Tangible magnetic analogy to computational simulation (quantum entanglement)	Constructivist scaffolding	Cognitive	Sequencing from concrete analogy to simulation addressed the limits of formalism-heavy approaches
105	Chen et al. (2025)	China	Lower sec.	Three-group quasi-experiment; 120 grade-9 students, three intact classes	Structured virtual lab (SIVL) in a flipped classroom	Structured inquiry	Cognitive; Affective; Inquiry skills	SIVL improved achievement, inquiry skills, and both intrinsic and extrinsic motivation
418	Coban et al. (2025)	Germany	Higher ed.	Experiment + eye tracking; Undergraduates in a university lab course	AR + personalised ChatGPT feedback (quantum cryptography)	Inquiry with AI-generated feedback	Cognitive; Learning process	AR visualised abstract concepts; personalised LLM feedback supported the cognitive learning process
597	Cook et al. (2022)	United States	Higher ed.	Naturalistic split-design study; 35 students per section, mechanics course	Video + virtual lab simulation (remote IBLA)	Inquiry	Cognitive; Affective	Students perceived the video-plus-simulation combination as more effective than either component alone
579	Evangelou and Kotsis (2019)	Greece	Primary	Randomised controlled pretest–posttest experiment; 110 grade-5 students (experimental 55, control 55; 58 boys, 52 girls)	Real vs virtual experiment (frictional force)	Experimental inquiry	Cognitive	Both modes were equally effective for building conceptual understanding of frictional force at primary level
774	Fan et al. (2018)	China	Upper sec.	Quasi-experiment, four classes; 117 students and 2 teachers, Beijing	Interactive simulations (ISIA)	Inquiry	Cognitive; SPS; Affective	ISIA superior on conceptual understanding, inquiry process skills, and confidence in learning
14	Faqih et al. (2023)	Indonesia	Upper sec.	R&D (ADDIE); 10 grade-11 students; 2 content experts, 2 media experts	Fisikawaii LMS: VR + gamification (GAFCC)	Inquiry-collaborative	Affective	Valid (content 85.5%; media 87.25%; student response 86.4%); learning participation increased
670	Fatmaryanti et al. (2022)	Indonesia	Higher ed.	R&D (modified Borg & Gall); 54 pre-service physics teachers, 5 lecturers	Virtual-lab task model	Inquiry + social and scientific communication	Inquiry skills; Collaborative	A nine-step task model proved essential for inquiry skills and for communication
86	Fatmaryanti et al. (2024)	Indonesia	Higher ed.	R&D (ADDIE) + one-group pretest–posttest DBR; Prospective physics teachers	Remote laboratory (2 experimental apparatuses)	Active and authentic learning	Cognitive	Remote-lab design improved prospective physics teachers' multiple-representation ability
223	Flegr et al. (2023a)	Germany	Lower sec.	Three-condition experiment; 154 grade-7 students analysed (159 recruited)	Virtual experiment + video recording	Inquiry	Cognitive	Inquiry improved conceptual understanding; virtual+video combination did not exceed virtual alone
425	Flegr et al. (2023b)	Germany	Lower sec.	Three-condition experiment; 202 students analysed (275 recruited, 11 classes, 4 schools)	Combination of real + virtual experiments	Inquiry	Cognitive	The real–virtual combination outperformed the single real experiment; sequence was not decisive
396	Ford et al. (2023)	United States	Higher ed.	Classroom study (analysis of 36 lab reports); 36 lab reports, mechanics-of-materials course	Remote laboratory + home kits	Collaborative guided inquiry	Affective; Cognitive	Goal-setting agency rose from 53% to 83%; 25 distinct design approaches
595	Fuhrmann et al. (2018)	United States	Upper sec.	Three-condition experiment (classes randomised); Grade-9 students	Bifocal Modelling: physical experiment + real-time computer modelling	Science inquiry	Cognitive; Metamodeling	Designing one's own model versus interacting with a given model produced different content and metamodeling outcomes
116	Fukuda et al. (2024)	Canada	Higher ed.	Randomised experiment, four groups; 83 undergraduates recruited, 80 analysed after 3 outliers were excluded (M age 19)	Electric-circuit simulation + on-demand domain text	Inquiry with just-in-time prompts	Cognitive	Just-in-time inquiry prompts facilitated rule learning, moderated by prior knowledge; self-explanation guidance showed no main effect
871	Gerhátová et al. (2020)	Slovakia	Primary	Two-group experiment (two-year study); 155 grade-9 students (experimental 85, control 70)	INTe-L: real, remote, and simulated experiments	Project-based	Cognitive	Project-based teaching with INTe-L was more effective than instructionist teaching
261	Gnesdilow and Puntambekar (2025)	United States	Lower sec.	Quasi-experiment + reasoning analysis; 385 students analysed of 552 (physical lab 180, virtual 205)	Physical vs virtual lab (pulley)	Collaborative inquiry	Cognitive; Transfer	Ability to apply learning to a new context differed between modes; apparatus complexity affected outcomes
668	Hamed and Aljanazrah (2020)	Palestine	Higher ed.	Mixed-methods quasi-experiment; 90 undergraduates (experimental 45, control 45)	Virtual experiments in a general physics lab	Inquiry	Cognitive; SPS	Virtual experiments were at least as effective as face-to-face theoretical preparation
622	Husnaini and Chen (2019)	Indonesia	Upper sec.	Quasi-experiment with random assignment; 68 secondary students	PhET (pendulum) vs technology-enhanced physical lab (Camera Stopwatch, Smart Tools)	Guided inquiry	Cognitive; Affective; Inquiry skills	Virtual lab superior on difficult concepts and inquiry self-efficacy; physical lab superior on planning and refining experiments
149	Jian et al. (2024)	Taiwan	Higher ed.	Experiment; 2×3 factorial ANOVA + sequential analysis; 105 undergraduates (hands-on 53, simulated 52)	Simulated vs hands-on experiment (pulley mechanics) + eye tracking	Inquiry	Cognitive; Learning process	Gaze patterns and learning processes differed between modes; effects moderated by prior knowledge
549	Karelina et al. (2022)	United States	Higher ed.	Comparative survey (eight-channel flow model); Physics undergraduates, ISLE design labs	Apparatus-based vs video-based lab	Design inquiry	Affective	The video group more often occupied the worry and anxiety zones, calling for different scaffolding
336	Karim et al. (2025)	Malaysia	Upper sec.	Mixed-method case study; 15 urban upper-secondary students	AR + scaffolding kit (gravitation)	Scaffolding within inquiry	Cognitive; HOT skills	Integrating scaffolding with AR improved problem solving involving mathematical calculation
665	Lämsä et al. (2022)	Finland	Higher ed.	Multimodal study (video + eye tracking, ENA); Two pairs of physics undergraduates	Simulation-based environment (electrostatics, via Zoom)	Collaborative knowledge construction	Collaborative	The focus and timing of joint gaze marked the level of collaborative knowledge construction
810	Mihret et al. (2022)	Ethiopia	Higher ed.	Quasi-experiment, non-equivalent comparison group; 63 second-year pre-service physics teachers, three teacher colleges	Blended lab (virtual + real)	Experimental inquiry	Affective	Blended laboratory experimentation improved pre-service teachers' attitudes towards physics laboratories
281	Mihret et al. (2023)	Ethiopia	Higher ed.	Quasi-experiment, non-equivalent comparison group; 63 pre-service physics teachers (blended 16, virtual 26, real 21)	Blended, virtual, and real physics lab (electricity and magnetism)	Experimental inquiry	Cognitive (NOS)	Blended laboratory experimentation was most effective for understanding the nature of science
436	Mufit et al. (2025)	Indonesia	Upper sec.	Development research + limited trial; Upper-secondary students	AR with a cognitive-conflict model + STEM integration	Cognitive conflict	Cognitive (sci. literacy)	AR-supported practical learning supported scientific literacy on Newton's law of gravitation
473	Narayanan et al. (2023)	India	Higher ed.	Multi-year implementation case study; 60 undergraduates at a leading Indian technological university	ESSENCe electronics lab (staging, scaffolding, instruments)	Collaborative inquiry	Inquiry skills; Collaborative	The ESSENCe framework trained experimental research skills through scaffolding and collaboration
407	Ouahi et al. (2026)	Morocco	Upper sec.	Two-group quasi-experiment; 35 students in the comparison group	Interactive simulations	5E learning cycle	Affective; Cognitive	Attitude towards physics shaped performance; interactive simulations improved both
749	Oymak and Ogan-Bekiroglu (2021)	Türkiye	Upper sec.	Experiment with random assignment; 96 grade-9 students (48 per group)	Virtual vs physical manipulatives (kinematics, dynamics)	Inquiry	Cognitive; Affective	Effects equivalent; virtual manipulatives marginally advantageous because they avoid student measurement error
15	Papalazarou et al. (2024)	Greece	Upper sec.	Quasi-experiment; 38 grade-3 upper-secondary students (20 vs 18)	Virtual vs physical lab (mechanics, electricity)	Inquiry	Cognitive; Affective	No significant difference between modes; attitudes equally positive
362	Pavlou et al. (2024)	Cyprus	Primary	Comparative experiment across domains; Preschool children	Physical vs virtual manipulatives	Experimental inquiry	Cognitive	Haptic feedback was not always decisive; the advantage of each mode depended on the science domain
535	Puntambekar et al. (2021)	United States	Lower sec.	Comparative study of group discourse; 115 grade-6 students (virtual 61, physical 54; 14 groups recorded)	Physical vs virtual lab	Collaborative inquiry	Collaborative	Virtual labs elicited more talk about predictions and variable relationships; physical labs more procedural talk
807	Puspitaningtyas and Putri (2021)	Indonesia	Upper sec.	One-group pretest–posttest mixed methods; A group of upper-secondary students	Virtual laboratory on light waves	Structured inquiry	Cognitive	Mastery of wave-optics concepts improved; interference and diffraction remained difficult
90	Rizki et al. (2025)	Indonesia	Upper sec.	Three-group quasi-experiment; 102 students (ESIL 35, ordinary inquiry 34, traditional 33)	EthnoVLab: ethnoscience virtual simulation + AR	Inquiry	Affective; HOT skills	ESIL group outperformed ordinary inquiry and traditional instruction on creativity and motivation
528	Roll et al. (2018)	Canada	Higher ed.	Two-condition experiment; 97 undergraduates (non-directive 49, directive 48)	PhET Circuit Construction Kit	Inquiry with varied guidance	Cognitive; Affective; Behaviour	Directive guidance raised knowledge for the higher-knowledge group but suppressed their attitudinal growth
61	Saprudin et al. (2025)	Indonesia	Higher ed.	Three-group experiment; 54 prospective teachers, Basic Physics Experiment I course, Ternate	AR within the URSLA framework	Inquiry (research skill development)	Cognitive; SPS	URSLA-AR group outperformed URSLA without AR and conventional instruction on science competencies
884	Sari et al. (2019)	Türkiye	Higher ed.	Pretest–posttest quasi-experiment; 60 undergraduates	Virtual lab vs computer-based real lab	Experimental inquiry	Affective; SPS	Computer-based lab superior on graphing skills; attitudes and motivation equivalent
419	Sebastian et al. (2023)	Indonesia	Upper sec.	One-group pretest–posttest quasi-experiment; 61 grade-11 science students from two classes	Flip-book + virtual lab simulation (optical instruments)	PBL	Cognitive	Visual representation improved; N-gain 0.828 (high category)
233	Siantuba et al. (2023)	Zambia	Higher ed.	Quasi-experiment; 171 first-year undergraduates (experimental 100, control 71)	Online digital laboratory (Go-Lab)	Misconception-targeted inquiry	Cognitive	Inquiry activities designed to target misconceptions produced greater conceptual change
363	Sinensis et al. (2025)	Indonesia	Higher ed.	Pretest–posttest pre-experiment; 67 pre-service physics teachers	AR-based teaching materials (modern physics) + mind mapping	Collaborative problem solving	HOT skills	Creativity increased significantly (p = 0.001; Rasch analysis and paired-samples test)
683	Taibu et al. (2021)	United States	Higher ed.	Mixed methods (one-semester group project); 61 participants across two semesters	PhET in group projects	Collaborative inquiry	SPS; Affective	Large effect size on laboratory skills (d > 0.8); 88% positive responses
724	Taşlıdere (2021)	Türkiye	Higher ed.	Comparative quasi-experiment; Pre-service science teachers	Simulation activities + concept-cartoon conceptual change texts	5E learning cycle	Cognitive	The 5E model with simulations and conceptual change texts both improved understanding of mechanical waves
672	Thees et al. (2022)	Germany	Higher ed.	Randomised experiment, two conditions; Undergraduates (AR condition N = 49; 80% male, M age 19)	AR head-mounted display vs separate display during lab work	Hands-on inquiry + multimedia	Cognitive	Spatial contiguity through AR affected coherence formation and cognitive load
179	Tokatlidis et al. (2024)	Greece	Higher ed.	Two-group quasi-experiment + ANCOVA; 152 undergraduates (136 male, 16 female; aged 19–21)	TINA-TI virtual lab vs hands-on lab (operational amplifiers)	Inquiry	Cognitive; Inquiry skills; Measurement skills	Hands-on group outperformed the virtual group (F = 9.04; p = 0.003); knowledge gains largest, then inquiry skills
740	Tsivitanidou et al. (2021)	Cyprus	Upper sec.	Quasi-experiment + cluster analysis; 107 upper-secondary students	Immersive VR (special theory of relativity)	Inquiry	Cognitive; Affective	Significant interaction between conceptual gains and learners' pre-existing attitudinal profiles
194	Warkentin et al. (2026)	Germany/Switzerland	Higher ed.	Design research (learners not yet tested); —	AR smartglasses (Lorentz force)	Hands-on inquiry + multiple representations	Cognitive (design)	Superimposing fields in AR linked phenomenon to representation and reduced cognitive load
348	Xing et al. (2023)	United States	Upper sec.	Design-based research + learning analytics; Grade-9 students	Infrared Explorer: physical experiment + virtual environment (thermodynamics)	Inquiry with concreteness fading	Cognitive	Augmented interaction strengthened thermodynamics understanding; learning analytics mapped the process
394	Yakkou et al. (2024)	Morocco	Upper sec.	Quasi-experiment; 35 students	Electrical-systems evolution simulator	Inquiry (prediction, variable control, hypothesising)	Affective; Cognitive	The simulator improved motivation and academic performance in electricity lessons
697	Yuliati et al. (2018)	Indonesia	Higher ed.	Mixed methods, embedded experimental model; 34 pre-service physics teachers, Jambi State University	PhET (direct-current electricity)	Inquiry	Cognitive; HOT skills	Students using a scientific approach showed better problem-solving skills
687	Yusuf and Widyaningsih (2020)	Indonesia	Higher ed.	Qualitative study through lesson-study stages; Physics Education students, University of Papua	E-learning-based virtual laboratory media	Inquiry within lesson study	Metacognitive	E-learning-based virtual lab media fostered students' metacognitive skills
322	Yusuf et al. (2024a)	Indonesia	Higher ed.	R&D (ADDIE); 54 undergraduates, University of Papua	Virtual laboratory platform (Physics Fun)	POGIL	Cognitive; Acceptance	Media effective and practical; experimental capability improved in remote and underserved regions
108	Yusuf et al. (2024b)	Indonesia	Upper sec.	Quantitative survey (SEM; TAM–TRA); 136 upper-secondary students, West Papua	Mobile V-Lab (Physics Fun)	POGIL	Technology acceptance	Attitude and behavioural intention driven by perceived ease of use and subjective norm; perceived usefulness had no direct effect

SPS = science process skills; HOTS = higher-order thinking skills. The ID column is the record number in the database export, retained so that each study can be traced to the selection audit trail in the extended data. Mihret et al. (2022) and Mihret et al. (2023) are two reports of one study. An em rule marks information not reported in the source article.

**Table 5.** Item-level MMAT 2018 appraisal of all 59 included reports.

*Category 3. Quantitative non-randomised * (n = 23 reports)							
ID	Study	3.1 Participants representative	3.2 Measurements appropriate	3.3 Complete outcome data	3.4 Confounders accounted for	3.5 Intervention as intended	Criteria met
118	Abuna et al. (2026)	not met	met	met	not met	not met	2 of 5
309	Alabidi et al. (2023)	not met	met	met	not met	cannot tell	2 of 5
451	Banda and Nzabahimana (2023)	met	met	met	met	cannot tell	4 of 5
464	Ben Ouahi et al. (2024)	cannot tell	met	met	met	cannot tell	3 of 5
105	Chen et al. (2025)	met	met	met	met	cannot tell	4 of 5
597	Cook et al. (2022)	cannot tell	not met	not met	not met	met	1 of 5
774	Fan et al. (2018)	cannot tell	met	cannot tell	met	met	3 of 5
871	Gerhátová et al. (2020)	not met	met	met	not met	met	3 of 5
261	Gnesdilow and Puntambekar (2025)	cannot tell	met	not met	met	met	3 of 5
622	Husnaini and Chen (2019)	cannot tell	met	met	met	not met	3 of 5
149	Jian et al. (2024)	cannot tell	met	met	met	met	4 of 5
810	Mihret et al. (2022)	not met	met	met	not met	met	3 of 5
281	Mihret et al. (2023)	not met	met	met	met	cannot tell	3 of 5
407	Ouahi et al. (2026)	cannot tell	met	met	met	met	4 of 5
90	Rizki et al. (2025)	cannot tell	met	met	not met	met	3 of 5
61	Saprudin et al. (2025)	cannot tell	met	met	not met	cannot tell	2 of 5
884	Sari et al. (2019)	not met	met	met	not met	not met	2 of 5
233	Siantuba et al. (2023)	cannot tell	met	met	met	cannot tell	3 of 5
724	Taşlıdere (2021)	not met	met	met	met	met	4 of 5
179	Tokatlidis et al. (2024)	cannot tell	met	met	met	met	4 of 5
740	Tsivitanidou et al. (2021)	not met	met	met	not met	met	3 of 5
348	Xing et al. (2023)	cannot tell	not met	not met	not met	met	1 of 5
394	Yakkou et al. (2024)	cannot tell	not met	cannot tell	met	met	2 of 5

*Category 2. Quantitative randomised controlled trials* (n = 12 reports)							
ID	Study	2.1 Randomisation performed	2.2 Groups comparable at baseline	2.3 Complete outcome data	2.4 Assessors blinded	2.5 Adherence to intervention	Criteria met
418	Coban et al. (2025)	cannot tell	met	met	not met	not met	2 of 5
579	Evangelou and Kotsis (2019)	met	met	met	cannot tell	met	4 of 5
223	Flegr et al. (2023a)	cannot tell	met	not met	cannot tell	met	2 of 5
425	Flegr et al. (2023b)	met	met	not met	met	met	4 of 5
595	Fuhrmann et al. (2018)	cannot tell	cannot tell	met	cannot tell	cannot tell	1 of 5
116	Fukuda et al. (2024)	cannot tell	met	met	cannot tell	met	3 of 5
749	Oymak and Ogan-Bekiroglu (2021)	cannot tell	met	not met	not met	met	2 of 5
15	Papalazarou et al. (2024)	cannot tell	not met	not met	met	not met	1 of 5
362	Pavlou et al. (2024)	cannot tell	met	met	not met	met	3 of 5
535	Puntambekar et al. (2021)	met	cannot tell	not met	cannot tell	met	2 of 5
528	Roll et al. (2018)	met	met	met	cannot tell	met	4 of 5
672	Thees et al. (2022)	cannot tell	met	met	cannot tell	met	3 of 5

*Category 4. Quantitative descriptive* (n = 14 reports)							
ID	Study	4.1 Sampling strategy relevant	4.2 Sample representative	4.3 Measurements appropriate	4.4 Nonresponse bias low	4.5 Analysis appropriate	Criteria met
164	Auliya et al. (2025)	met	not met	cannot tell	cannot tell	met	2 of 5
925	Bahtiar et al. (2022)	met	not met	met	cannot tell	not met	2 of 5
14	Faqih et al. (2023)	met	not met	cannot tell	cannot tell	met	2 of 5
670	Fatmaryanti et al. (2022)	cannot tell	not met	cannot tell	cannot tell	met	1 of 5
86	Fatmaryanti et al. (2024)	cannot tell	not met	met	cannot tell	met	2 of 5
396	Ford et al. (2023)	met	not met	met	not met	met	3 of 5
549	Karelina et al. (2022)	met	not met	met	not met	not met	2 of 5
436	Mufit et al. (2025)	met	not met	cannot tell	cannot tell	cannot tell	1 of 5
419	Sebastian et al. (2023)	met	not met	cannot tell	cannot tell	not met	1 of 5
363	Sinensis et al. (2025)	met	not met	not met	cannot tell	met	2 of 5
194	Warkentin et al. (2026)	cannot tell	not met	met	cannot tell	met	2 of 5
687	Yusuf and Widyaningsih (2020)	cannot tell	not met	cannot tell	met	not met	1 of 5
322	Yusuf et al. (2024a)	cannot tell	not met	met	cannot tell	met	2 of 5
108	Yusuf et al. (2024b)	cannot tell	not met	met	met	met	3 of 5

*Category 5. Mixed methods* (n = 8 reports)							
ID	Study	5.1 Rationale for the design	5.2 Components integrated	5.3 Integration interpreted	5.4 Divergences addressed	5.5 Each strand meets its tradition	Criteria met
191	Adimayuda et al. (2025)	met	cannot tell	cannot tell	met	not met	2 of 5
668	Hamed and Aljanazrah (2020)	met	met	met	met	not met	4 of 5
336	Karim et al. (2025)	met	not met	not met	cannot tell	not met	1 of 5
665	Lämsä et al. (2022)	met	met	met	cannot tell	cannot tell	3 of 5
473	Narayanan et al. (2023)	met	cannot tell	cannot tell	cannot tell	not met	1 of 5
807	Puspitaningtyas and Putri (2021)	cannot tell	met	met	not met	not met	2 of 5
683	Taibu et al. (2021)	met	cannot tell	not met	cannot tell	not met	1 of 5
697	Yuliati et al. (2018)	cannot tell	met	met	cannot tell	not met	2 of 5

*Category 1. Qualitative* (n = 2 reports)							
ID	Study	1.1 Approach appropriate	1.2 Data collection adequate	1.3 Findings derived from data	1.4 Interpretation substantiated	1.5 Coherence across analysis	Criteria met
182	Alstein et al. (2026)	met	met	met	met	met	5 of 5
430	Castillo-Salazar et al. (2026)	met	met	not met	not met	not met	2 of 5

Each report is judged only against the five criteria of its own design category, so counts are not comparable across categories. Answers are met, not met, or cannot tell, the last recording that the report gives too little information to judge. Following the guidance of the instrument developers, no composite score or quality ranking was computed from these answers. The justification and evidence page behind every answer are in the appraisal workbook in the extended data.

### 2.2 Sources and search

A literature search was conducted on July 9, 2026, using the Scopus database. This database was selected on the basis of its track record and comprehensive coverage of reputable science education journals. No register, gray literature, or citation tracking was searched. The strategy combined a domain block, pedagogy block, and technology block with Boolean operators, limited to 2017 to 2026, and journal articles and conference papers, with no language limiter. Appendix A provides details of the search syntax. A post-export evaluation showed that the query covered a broader search area than titles, abstracts, and keywords. Consequently, the search results exhibited a high level of sensitivity, but low precision. The numerical details related to this finding are outlined in Appendix A, and the large exclusion counts are explained in Figure 1.

**Figure 1.**
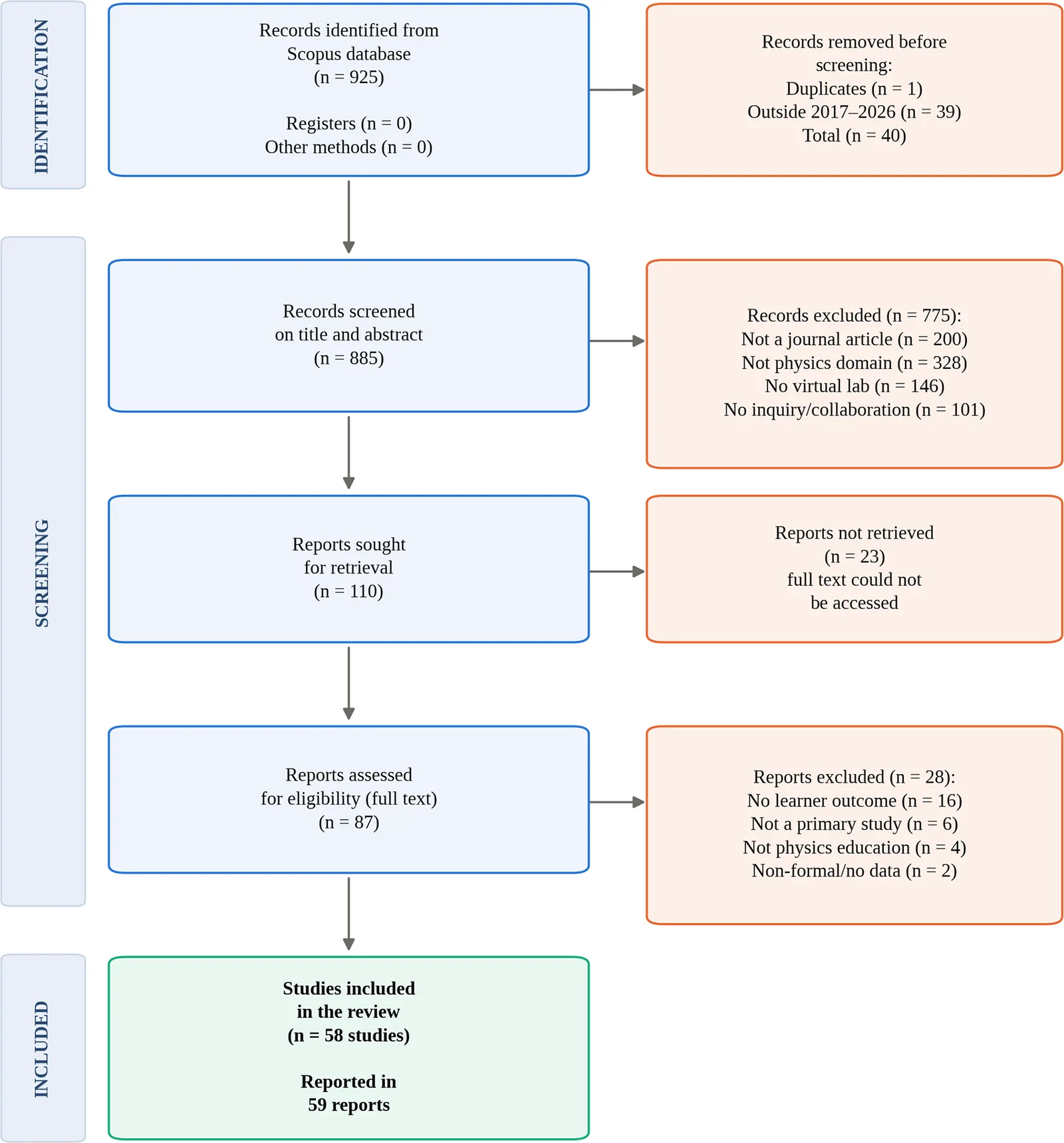
PRISMA 2020 flow of the study selection process. Template A, adapted from Page et al. (2021). *Records* are database entries, *reports* full-text documents, and *studies* the underlying investigations; two reports describe one study, so 59 reports give 58 studies.

### 2.3 Selection

All retrieved records were parsed, filtered by publication year, de-duplicated on the digital object identifier, and then on normalized titles. Titles and abstracts were screened through four sequential stages: document type, physics domain, virtual laboratory component, and the application of an inquiry or collaborative model using a controlled term dictionary. Every exclusion was logged together with the stage that produced it; therefore, all 925 records remained traceable.

All the selection decisions were then manually audited against the criteria listed in Table 1. This screening process took place in two rounds, with the second round using a more extensive list of terms. Its impact on the data corpus is described in Section 4.2. Twenty-three reports were eligible but could not be downloaded because of a paywall. Following PRISMA 2020, these are reported as reports not retrieved rather than as records excluded against the eligibility criteria; Appendix C lists them.

The initial screening and eligibility evaluations were conducted by the first author. Each recorded decision was independently re-evaluated by four co-authors. They reviewed the documents based on the available notes and reasons for rejection and highlighted any decisions they deemed inappropriate. In both stages of the selection process, the literature marked and those that failed to be determined as eligible by the first author were forwarded to the second and third authors. Any disagreements were resolved through discussion until a consensus was reached, with no single reviewer having a casting vote. The final agreed-upon decision was documented in the selection log.

### 2.4 Extraction and quality appraisal

Data were extracted from the full text of each report by using a structured form with fixed fields. The extracted items were: study identity, country, educational level, design, sample, technology type, learning model, physics topic, and reported outcomes. The reported outcomes were classified into eight competency categories: conceptual understanding and achievement, attitude, motivation, self-efficacy, inquiry skills, science process skills, collaboration and communication, higher-order thinking, metacognition and self-regulation, and technology acceptance. Both the extraction of study characteristics and item-level Mixed Methods Appraisal Tool (MMAT) judgements were produced with the assistance of a generative artificial intelligence tool while reading the full texts. The first author checked every judgement against the source report before reporting it. Items that were not reported explicitly were recorded as unavailable or not imputed. Full-text reading also showed that Mihret et al. (2022) and Mihret et al. (2023) reported the same study of 63 pre-service teachers in the same electricity and magnetism laboratory course, with different dependent variables. Following PRISMA 2020 items 16a and 17, they are counted as two reports of one study; therefore, the corpus contains 58 studies reported in 59 reports.

Methodological quality was assessed using the Mixed Methods Appraisal Tool (MMAT), version 2018 ( Hong et al., 2018), which covers quantitative, qualitative, and mixed designs within a single framework. Each report was assigned to one of the five MMAT design categories and judged against the five criteria of that category as met, not met, or unable to tell, and the last recording that the report gave too little information to judge. A report whose authors described it as quasi-experimental and whose randomization procedure is unreported was appraised as non-randomized, even where random allocation of intact classes is mentioned. Following the developers' advice, no composite score or quality ranking was calculated; instead, the item-level profile was reported, and the full grid is shown in Table 5.

### 2.5 Synthesis

Studies were grouped by instructional design configuration rather than by physics topic because the research questions target the relationship between pedagogy and technology and were tabulated in an extraction matrix ( Table 4). Synthesis followed a six-phase thematic analysis ( Braun & Clarke, 2006), coding inductively from reported findings, and then reviewing the codes towards the inquiry ( Pedaste et al., 2015) and collaboration ( Chi & Wylie, 2014) frames. The synthesis is narrative throughout: the interventions, instruments, and designs vary too widely for statistical pooling; therefore, effect sizes reported by the primary studies, chiefly Cohen's d and N-gain, were recorded as given and read as markers of magnitude within each study. Robustness was examined by asking whether each theme was held when the synthesis was restricted to reports meeting at least three of the five MMAT criteria, following the developers' guidance, served only as a filter for that check, and was not reported as a quality score or ranking. Reporting bias was assessed narratively through the proportion of null and counter-directional findings, match between stated aims and reported outcomes, database and language restrictions, and unretrieved reports. Certainty in each theme was rated as high, moderate, or low using four considerations adapted from GRADE: number of supporting studies, consistency of direction, methodological quality, and transferability.

## 3. Results

### 3.1 Study selection

The search returned 925 records, of which 40 were removed before screening: 39 outside the publication window and one duplicate. Of the 885 records screened on title and abstract, 775 were excluded: 200 were not journal articles, 328 lay outside physics or physical science, 146 carried no virtual laboratory or simulation components, and 101 applied neither an inquiry nor a collaborative model. Of the 110 reports, 23 could not be accessed. Eighty-seven were assessed in full text and 28 were excluded for the following reasons: 16 reported no learner outcome, six were not primary studies, four were set outside physics education, and two were in non-formal contexts or with no empirical learner data. Fifty-nine reports met the criteria, two described one study, and 58 studies. Figure 1 illustrates this process. All included reports are shown in Table 4 and in the reference list.

### 3.2 Characteristics and methodological quality of the evidence (RQ1)

The corpus covered 22 countries. Indonesia has the largest share, with 17 reports (28.8%), followed by the United States (n = 8) and Germany (n = 4, with one further German-Swiss collaboration), followed by Greece, Ethiopia, Morocco, and Türkiye, with three each. Participants were most often in higher education (n = 27), mainly in teacher preparation, upper secondary school (n = 22), lower secondary (n = 7), and primary school (n = 3). Figure 2 shows both the distributions.

**Figure 2.**
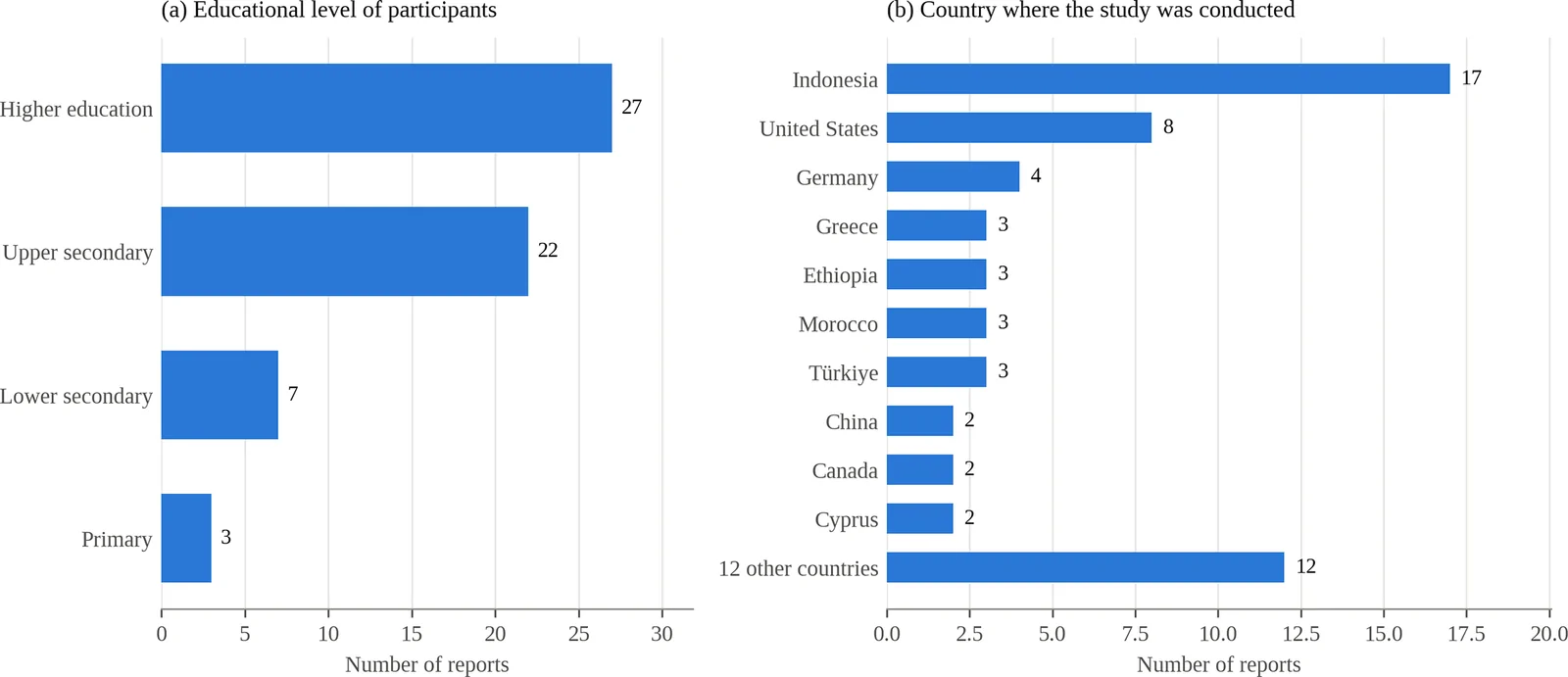
Research samples by educational level and country. n = 59 reports. Panel (b) shows the ten largest contributors; the remaining 12 countries contributed one report each.

Publication grew in the second half of the window: 16 reports appeared between 2018 and 2021, and 43 between 2022 and 2026, peaking in 2023 (n = 11). No 2017 reports entered the corpus; the two that met the criteria were among the reports not retrieved and are listed in Appendix C. Quasi-experimental designs dominated with 21 reports, followed by experiments without explicit randomization (n = 9), design and development research (n = 8), process or qualitative analysis (n = 8), randomized experiments (n = 5), mixed methods (n = 4), and two each for surveys and pre-experiments ( Figure 3).

**Figure 3.**
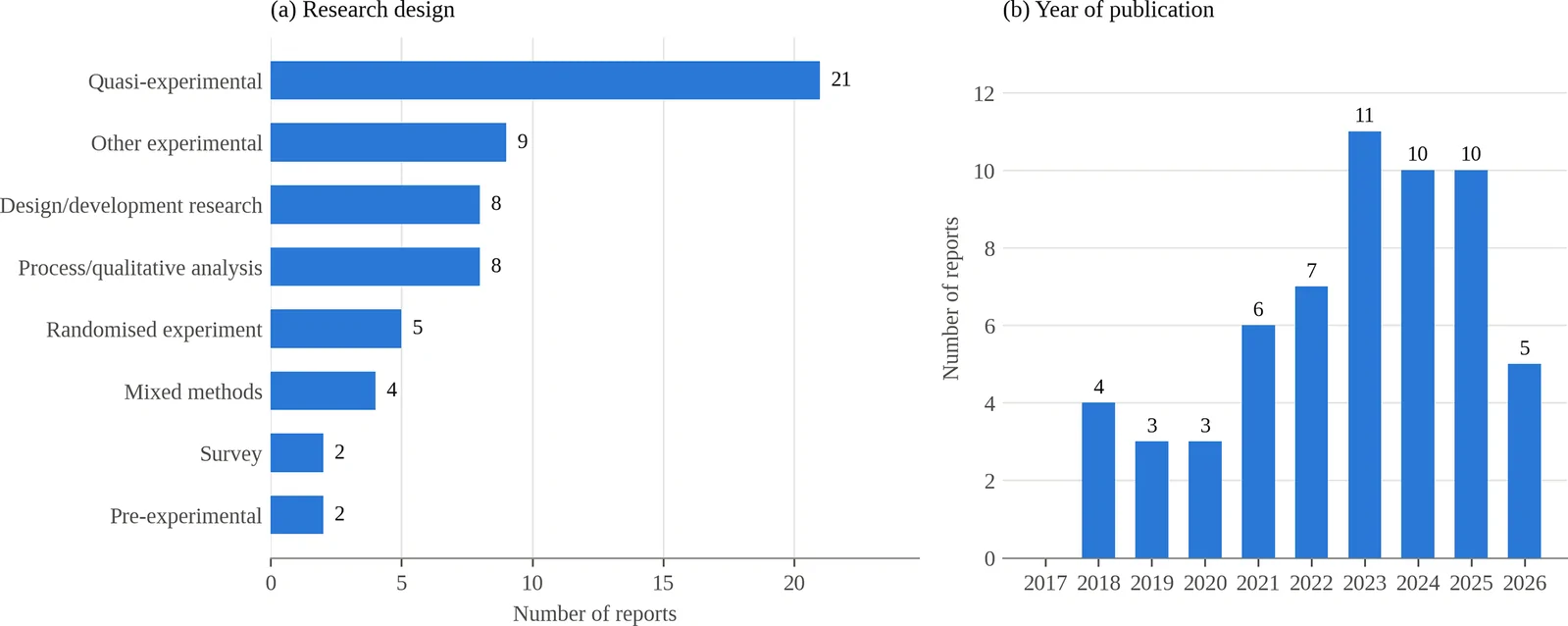
Research designs and publication years. n = 59 reports.

Under the MMAT, the corpus is divided into 23 non-randomized quantitative studies, 14 quantitative descriptive studies, 12 randomized trials, eight mixed-methods studies, and two qualitative studies. Table 2 lists the criteria most and least often met in each category, and Table 5 lists the full grid. The profile points to reporting and not conducting it. Measurement is the strongest area: instruments were appropriate in 20 of 23 non-randomized studies, most used validated concept inventories or tiered diagnostic tests, and outcome data were complete in 18. The weakest is the warrant for generalization, since almost no study argued that its sample represented a target population, whereas the rest drew on intact convenience classes. Procedural reporting is the second weakness: the randomization procedure was described in four of 12 randomized trials and blinding of outcome assessors in two. Confounders were controlled statistically, usually by covariance analysis on a pretest, in 13 of the 23 non-randomized studies; the other ten reported a pretest without adjusting for it.

### 3.3 Configurations of pedagogy and technology (RQ2)

Figure 4 maps learning models against laboratory technologies. Web-based virtual laboratories and interactive simulations are the most common technology (n = 38), followed by augmented reality and PhET simulations with seven reports each, remote laboratories or video-recorded experiments (n = 5), and immersive virtual reality (n = 2). On the pedagogical side, 40 reports applied guided or structured inquiry, eight an inquiry-collaborative model or collaborative problem solving, three scaffolded inquiry, three the 5E learning cycle, two problem-based or project-based learning, two POGIL, and one discovery learning.

**Figure 4.**
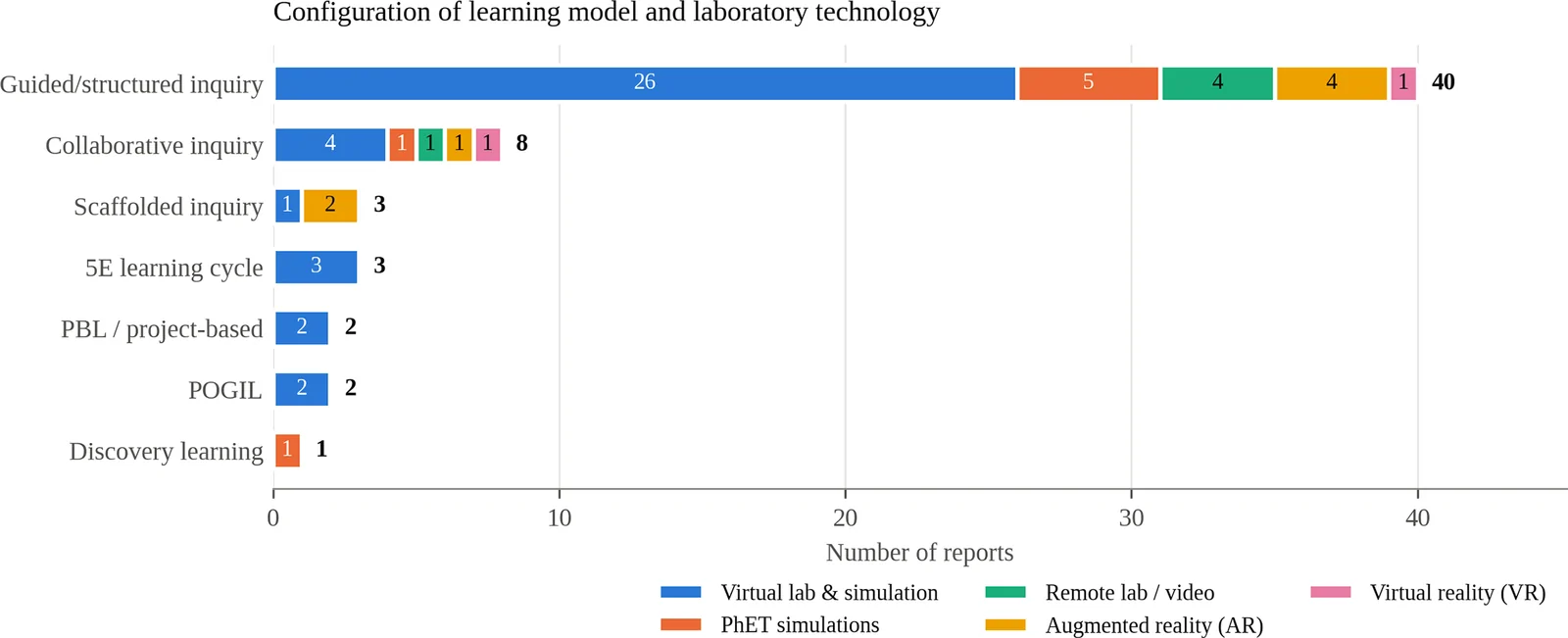
Configurations of learning model and laboratory technology. Each report is mapped to one dominant learning model and one dominant technology; bold figures give the total per model.

The configuration data answer RQ2 with asymmetry that runs through the entire review. Inquiry is an explicit, measured framework in almost every report, while collaboration is not. Although laboratory work almost always took place in groups, only nine of the 59 reports (15.3%) treated collaboration as an explicit design component through role structures, collaboration scripts, positive-interdependence tasks, or a collaborative problem-solving model, and only five (8.5%) measured a collaborative or communicative outcome. Elsewhere, group work is an unanalyzed setting, so its contribution cannot be separated from that of the technology or the inquiry model. This proportion is not an artifact of how the corpus is assembled. The 37 reports found by the first screening pass and the 22 recovered by the subsequent audit, which used a differently constructed and wider term list, gave almost the same figures: collaboration was designed explicitly in 16.2% and 13.6% of them, and measured in 8.1% and 9.1%, respectively.

### 3.4 Reported outcomes and their direction (RQ3)

Figure 5 shows the competencies measured. Conceptual understanding and achievement were dominant as measured in 45 studies (76.3%). Attitude, motivation, and self-efficacy were observed in 18 studies (30.5%), followed by science process skills (n = 6, 10.2%), inquiry skills, collaboration, and communication (n = 5 each, 8.5%), higher-order thinking (n = 4, 6.8%), metacognition and self-regulation (n = 3, 5.1%), and technology acceptance (n = 2, 3.4%). Competencies most often promised in the rationale for inquiry-based learning are the least measured.

**Figure 5.**
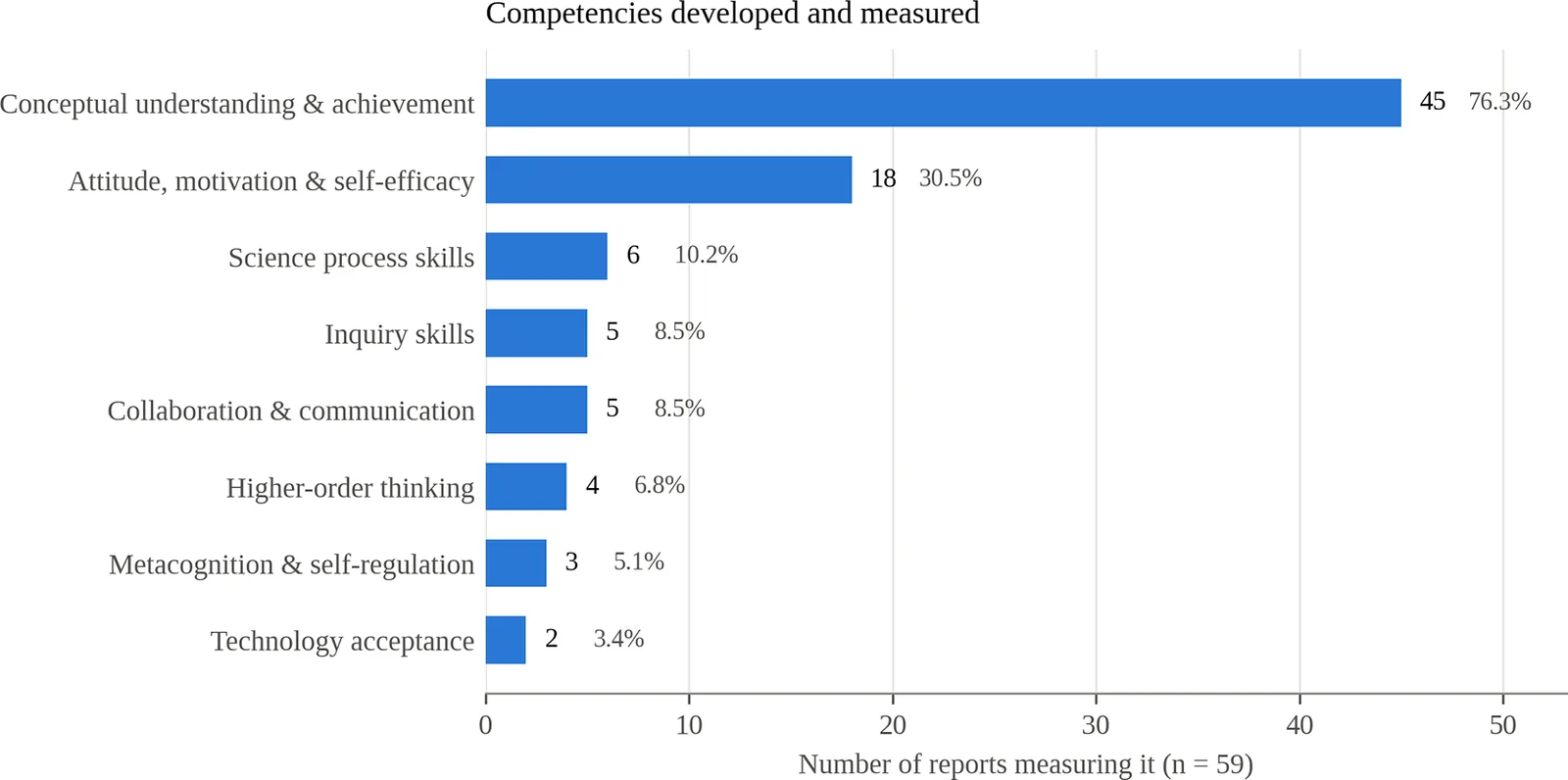
Competencies measured in the included studies. A study may measure more than one category, so the totals exceed 59. Percentages are of 59 reports.

Regarding conceptual outcomes, the evidence is separated into two layers. When one modality is compared with another, the equivalence dominates. Papalazarou et al. (2024) found no significant difference in conceptual understanding or attitudes among 38 upper secondary students. Evangelou and Kotsis (2019) found that the two modalities were equally effective for frictional force among 110 randomly assigned fifth-graders, and Oymak and Ogan-Bekiroglu (2021) obtained the same result with 96 ninth graders. The equivalence is conditional. Husnaini and Chen (2019) found a guided-inquiry virtual laboratory equal to a physical one on simple concepts but superior on difficult concepts and inquiry self-efficacy, while the physical laboratory was superior to inquiry activities requiring learners to plan and refine an experiment. In contrast, Tokatlidis et al. (2024) found a hands-on advantage in operational amplifier circuits, and Sari et al. (2019) found an advantage for computer-based over virtual laboratories in graph reading skills, so skills resting on instrument handling are not fully replaceable by simulation.

When modalities are combined, the direction becomes far more consistent. Flegr et al. (2023b) showed that real and virtual experiments together outperform real experiments alone among 202 secondary students, while Ben Ouahi et al. (2024) reported the largest electricity gains for a real-plus-virtual combination within a 5E cycle across 166 students in three conditions, and Mihret et al. (2023) found a blended modality most effective for understanding the nature of science. The recurring explanation is complementarity: real experiments supply the phenomenological referent, and simulations supply representations such as force vectors and dynamic graphs that physical apparatus cannot show. One exception bounds the claim: Flegr et al. (2023a) found no advantage for virtual experiments plus video over virtual experiments alone; therefore, the benefit depends on whether the combined components contribute complementary information, not simply more of it.

The affective results are further divided. Rizki et al. (2025) found an ethnoscience-based virtual simulation with augmented reality within an inquiry frame outperforming both plain inquiry and conventional teaching on creativity and motivation. Sinensis et al. (2025) reported significant creativity gains among 67 prospective teachers after collaborative problem solving with augmented reality material. Karelina et al. (2022) found that students working with video-recorded experiments occupy the worry and anxiety zones of a flow model more often than students using physical apparatus, and Tsivitanidou et al. (2021) added that learners' initial attitude profiles interact significantly with the conceptual gains they draw from immersive virtual reality, so the affective effect of a technology cannot be treated as a single effect that applies uniformly to all learners. Higher-order and metacognitive outcomes remain rare but promising: Adimayuda et al. (2025) reduced misconceptions of momentum and impulse in the high category by combining simulations, video, and an artificial-intelligence-assisted reflection tool, and Yusuf and Widyaningsih (2020) report that e-learning-based virtual laboratory media fostered the metacognitive skills of prospective teachers.

### 3.5 Moderating factors and implementation constraints (RQ4)

The clearest moderator is the degree and type of guidance, stated explicitly in 17 reports and manipulated directly in several reports. Roll et al. (2018) showed that with 97 undergraduates, directive guidance increased knowledge gains for learners with high prior knowledge while suppressing their attitude growth, whereas for learners with a high sense of competence, the same guidance left knowledge unchanged but promoted attitude growth. Fukuda et al. (2024) found that just-in-time inquiry prompts facilitate rule learning with the effect moderated by prior knowledge, while principle-based self-explanation guidance produced no main effect. Chen et al. (2025) showed that embedding a whole inquiry cycle into the virtual laboratory, instead of using the laboratory as one tool inside an inquiry process, produced simultaneous gains in achievement, motivation, and inquiry skills among 120 ninth-graders. Failures are also documented: Alstein et al. (2026) found students unable to interpret relativity simulations correctly even when the simulations did provoke cognitive conflict, and Thees et al. (2022) showed that presenting virtual information through a head-mounted display instead of a separate screen changes coherence formation and cognitive load.

The second moderator is how collaboration is structured, which is visible only in the few studies that have analyzed it. Puntambekar et al. (2021) analyzed the discourse of 14 groups from 115 sixth-graders and found that students in physical laboratories talk more about apparatus setup and computation, while students in virtual laboratories make more predictions and interpret relationships between variables more often, which shows that equivalence in test scores can conceal large differences in the learning process. Gnesdilow and Puntambekar (2025) extended this to 385 students and found that transfer to new contexts also differed by modality and that simplifying the complexity of physical apparatus changes what students learn. Lämsä et al. (2022) added micro-level evidence, combining video and eye-movement data from pairs of physics undergraduates to solve electrostatic problems in a simulation-based environment. The focus and timing of joint gaze marked the level of collaborative knowledge construction, whether new ideas, explication, or evaluation, which makes productive collaboration distinguishable from parallel work that merely proceeds side by side. Two studies were answered at the design level. Narayanan et al. (2023) combined staging, scaffolding, embedded information sources, instruments, and collaboration in the ESSENCe framework for teaching experimental research skills to 60 undergraduates. Fatmaryanti et al. (2022) proposed a nine-step task model that separates investigative steps from steps of social and scientific communication.

Implementation constraints are contextual rather than technical constraints. Yusuf et al. (2024a, 2024b) implemented a POGIL-based virtual laboratory platform in the frontier, outermost, and disadvantaged regions of Indonesia, where learners gained experimental capability previously out of reach, and acceptance among 136 students turned on perceived ease of use and subjective norm, not perceived usefulness. Abuna et al. (2026) added three preconditions: wider adoption, teacher training in inquiry pedagogy, and infrastructure policies. The cost explicitly enters evidence. Husnaini and Chen (2019) designed a low-cost physical laboratory supported by a smartphone application as the comparator for their virtual laboratory, and Gerhátová et al. (2020) combined real experiments, remote experiments, and simulations in one integrated strategy for 155 students in two Slovak primary schools. Thus, the practical choice is not between an expensive laboratory and a virtual one, but between designs that use the available resources well and those that do not. Hamed and Aljanazrah (2020) stated the position with the widest support: virtual experiments substitute best for theoretical preparation and complement the real laboratory rather than replace it.

### 3.6 Reporting bias and certainty of evidence

Several studies have reported state equivalence or a null result in their primary comparison: Papalazarou et al. (2024), Evangelou and Kotsis (2019), Oymak and Ogan-Bekiroglu (2021), and Hamed and Aljanazrah (2020). Others report findings that do not favor technology, including Tokatlidis et al. (2024) and Sari et al. (2019). Therefore, severe publication bias is less likely. Sources of bias remain: the single database and the English-language restriction; the concentration of intervention-favorable results among the reports meeting the fewest MMAT criteria; the under-reporting of collaborative outcomes relative to what the method descriptions imply; and the 23 reports that could not be obtained, most of them in subscription journals, so the synthesis may lean toward open-access publication.

Table 3 maps the five synthesis themes onto the research questions with their supporting evidence and certainty, which is high for the modality and guidance themes, which rest on 21 and 17 reports with consistent directions. Both also agree with meta-analytic evidence outside the corpus: the modality findings of de Jong et al. (2013) and Zacharia and de Jong (2014), and the guidance finding of Lazonder and Harmsen (2016). It is moderate for competency spread, where the affective direction is divided and instruments vary, and for contextual preconditions, which rest on a few context-specific studies. It is low for collaboration, not because the supporting evidence is weak, but because only five studies measured collaborative outcomes, too few to generalize; this finding is best read as a research gap. Restricting the synthesis to 27 reports meeting at least three of their five MMAT criteria left every theme intact in direction except collaboration, whose support fell from nine reports to three.

## 4. Discussion

### 4.1 Interpretation

Taken together, the 58 studies support one reading, and the value of the virtual laboratory lies in the pedagogical design around it rather than in technology. Three findings were obtained from this reading. Virtual and physical laboratories used alone are broadly equivalent for conceptual mastery, virtual mode gaining ground on abstract concepts and inquiry self-efficacy, and physical mode on skills resting on instrument handling. Combining the two consistently outperforms either alone, where the components contribute complementary information. Guidance based on prior knowledge determines whether exploratory freedom becomes a learning or cognitive load.

Equivalence on this scale extends de Jong et al. (2013) to a more recent and geographically broader body of work. More than a decade later, with mature simulation tools and augmented and virtual reality in classrooms, the pattern holds, which suggests that the advantages often claimed for new technology originate in the instructional redesign accompanying it, not in visual fidelity. The combination finding extends Zacharia and de Jong (2014): the benefit is representational rather than additive, which is consistent with multimedia learning theory ( Mayer, 2009). The guidance finding aligns with Lazonder and Harmsen (2016) and gives the dispute between Kirschner et al. (2006) and Hmelo-Silver et al. (2007), a specific empirical form in digital laboratories; the question is not whether to guide but how to fit guidance to prior knowledge and attitude profiles.

The most significant finding is negative. This review examines inquiry-collaborative models; however, these two components are not treated as equals in the literature. Inquiry is explicit and measured; collaboration is designed explicitly in 15.3% of the reports and measured in 8.5%. This gap matters because ICAP places the interactive mode at the highest level of engagement ( Chi & Wylie, 2014) and because computer-supported collaborative learning works through designed support ( Chen et al., 2018). The studies that analyzed the collaborative process showed equivalence in test scores concealing differences in group discourse, so the absence of collaborative measurement may lead the field to report equivalence where differences exist.

### 4.2 Limitations

The evidence is limited in four ways. Non-randomized designs dominated, and in ten of the 23 appraised under that category, a pretest was reported without being used to adjust for confounding, so teacher characteristics, time on task, and novelty remained uncontrolled. Almost no study has argued that its sample represents a target population, which limits generalization. Most interventions ran from one session to a few weeks with little delayed measurement, and many samples were small ( Table 4); therefore, novelty cannot be separated from durable learning. The instruments also vary widely.

This review process has its own limitations. The search covered one database and English-language publications, with no citation tracking or grey literature; the language restriction bears particularly here because Indonesia is the largest contributor, and much Indonesian physics education research appears in national journals outside Scopus. Initial screening used a controlled term dictionary that proved too narrow for the first application, omitting the 5E learning cycle and Process-Oriented Guided Inquiry Learning (POGIL), although Table 1 lists them as qualifying. A manual audit of all 925 decisions with a wider term list recovered 31 candidate reports, 22 of which were in the final corpus; therefore, more than a third of the included evidence was reached only on the second pass. Thus, the possibility of residual wrongful exclusions remains. A more fundamental gap sits in the search itself: the pedagogy block contained only inquiry and collaborative term variants and the technology blocked only laboratory term variants, so a study applying a 5E cycle with PhET simulations and never using phrase inquiry learning in an indexed field would never have entered the 925 records. Unlike the dictionary problem, no audit can repair this problem and its size cannot be estimated. Finally, 23 eligible reports (20.9% of those sought) could not be obtained; two measured the collaborative process. Finally, 23 eligible reports (20.9% of those sought) could not be obtained, two of which measured the collaborative process. Screening was carried out by the first author and then verified by co-authors working from the logged decisions, which is weaker than blind duplicate screening because the verifier sees the original judgement; no record was screened independently from scratch by two reviewers, so inter-rater agreement could not be calculated.

### 4.3 Implications

For teaching, three recommendations were made. Position the virtual laboratory as a complement to the real one, except where a real laboratory is out of reach, sequencing phenomenological experience with apparatus alongside representational exploration in simulation. Grade guidance by prior knowledge, giving novice worksheets, just-in-time prompts, and self-explanation questions while leaving advanced learners looser exploratory space, so their attitude growth is not suppressed. Design groups work deliberately, since productive collaboration depends on joint attention to a shared artifact, which makes role division and interdependent tasks matter to more than one device per student.

For policy, the geographic spread of the corpus shows virtual laboratories being used to equalize access to experimental work, but RQ4 evidence establishes that devices alone are insufficient. Improvement requires parallel investment in reliable infrastructure; teacher development focuses on inquiry pedagogy instead of software operation; and in validated worksheets, collaboration protocols, and assessment instruments. For research, the most pressing agenda is collaborative: studies that design collaboration explicitly and measure its outcomes and processes, following the precedents of Puntambekar et al. (2021) and Lämsä et al. (2022). Beyond that, the field needs longer randomized designs with delayed measurements, more work at the primary level, shared instruments for inquiry and collaborative skills, and reviews spanning more databases and languages.

## 5. Conclusions

This review of 58 studies reported in 59 reports published between 2018 and 2026 shows that the advantage of virtual laboratories in physics education comes from the quality of the pedagogical design around them rather than from the sophistication of the technology. Used alone, virtual and physical laboratories are broadly equivalent for conceptual mastery, the virtual mode being superior to abstract concepts and inquiry self-efficacy, and the physical mode of instrument-handling skills. Combining them consistently outperforms either alone, where the components contribute complementary information, and guidance graded to prior knowledge and attitude profile determines whether exploratory freedom aids learning or adds cognitive load.

Of the two pedagogical components examined in this review, only inquiry is treated explicitly and measured. Collaboration, though inherent in almost all laboratory work, was designed explicitly in 15.3% of reports and measured in 8.5%, and the same imbalance appeared across competencies, with conceptual understanding measured in 76.3% of studies, while collaboration and communication, inquiry skills, and higher-order thinking were each measured in fewer than a tenth. The evidence based on inquiry-collaborative models assisted by virtual laboratories is therefore still, in substance, evidence based on inquiry with virtual laboratories that happen to be delivered in groups. Bridging pedagogy and technology remains incomplete, while the social dimension of learning is treated as a setting rather than an object of design and measurement.

## Ethics and consent

The need for ethical approval and consent were not required. This study was a systematic review of previously published research and involved no human participants, human material, or personal data collected by the authors.

## Use of generative artificial intelligence

Generative artificial intelligence was used to prepare this review. Anthropic's Claude-opus-5 was used for two purposes: to write and revise the Python and JavaScript scripts that parsed the Scopus export, remove duplicates, apply the controlled screening term list, log every exclusion with the stage that produced it, and tabulate the extracted variables; and to produce the item-level judgements against the Mixed Methods Appraisal Tool 2018 criteria from the full texts of the included reports. The authors take full responsibility for the content of this article, including every judgement reported in Table 2 and 5, and for the accuracy of the data in the accompanying Zenodo deposit.

## Authors’ role in the peer review process

The authors understand that this article will undergo open, post-publication peer review, that the referee reports and referee names will be published alongside the article, that the authors are responsible for suggesting suitable referees, for responding publicly to each referee report, and for submitting a revised version where a report requires one.

## Data Availability

No new primary data were generated. The unit of analysis is the published report, and every included report is identified in the reference list and Table 4. The 925 Scopus records that entered screening, with the stage and reason recorded for every decision, were deposited as extended data so that the selection could be reproduced. Zenodo: Supporting data for 'Bridging Pedagogy and Technology: A Systematic Literature Review on Inquiry-Collaborative Models Assisted with Virtual Laboratory in Physics Education'. https://doi.org/10.5281/zenodo.22932800 ( Yahya et al., 2026). This project contains the following extended data:
• Extended_Data.docx. Appendix A, the search strategy with the full Boolean syntax and the post-export field-scope and precision check; and Appendix C, the 23 eligible reports sought, but not retrieved, with their identifiers. •
PRISMA_Screening_Audit_925_records.xlsx. The final selection log for all 925 records, with the stage, decision, reason, and evidence for each, is reconciled with Figure 1, which lists the 31 candidates recovered by the second screening pass with each record's first-pass decision (in Indonesian). •
Extraction_Matrix_59_reports.csv. The data in Table 4, with bibliographic fields and the screening pass through which each report entered the corpus in a machine-readable form (in Indonesian). •
MMAT_Appraisal_Grid_59_Reports.xlsx. The item-level appraisal grid is shown in Table 5, with the answer, justification, and evidence pages for each judgement. • scripts. Python scripts were used to parse the export, deduplicate, screen, audit the screening decisions, and tabulate the extracted variables. •
Figure1_PRISMA_flow.png — the PRISMA 2020 flow diagram ( Figure 1 of the article) • README.md. Description of each file with English translations of Indonesian column headings. Data are available under the terms of the Creative Commons Attribution 4.0 International licence (CC BY 4.0). No stand-alone software was developed for this study. The Python scripts used to parse the Scopus export, deduplicate, screen, audit the screening decisions, and tabulate the extracted variables are included in the Zenodo deposit under the MIT licence. They document the procedure and are provided for transparency. They are not standalone pipelines because they read intermediate files derived from Scopus exports that are not redistributed. The selection log records the outcome of each step for each record. Zenodo: PRISMA 2020 checklist and flow diagram for bridging pedagogy and technology: A systematic literature review on inquiry-collaborative models assisted with virtual laboratories in physics education. https://doi.org/10.5281/zenodo.22932800 ( Yahya et al., 2026). The review was reported in accordance with the Preferred Reporting Items for Systematic Reviews and Meta-Analyses (PRISMA) 2020 statement. The completed 27-item checklist, with the location of each item in this article, is given in Appendix B in the deposit above, and the flow diagram is shown in Figure 1. Data are available under the terms of the Creative Commons Attribution 4.0 International licence (CC BY 4.0). The review was not registered, and no protocol was published. A protocol covering the questions, eligibility criteria, search strategy, and synthesis method was prepared internally before the search was run; its substance is reported in Subsections 2.1 2.5.
