# Supplementary material for: Bridging Pedagogy and Technology: A Systematic Literature Review on Inquiry-Collaborative Models Assisted with Virtual Laboratory in Physics Education
Source: F1000Res. 2026 Oct 7;15:1682. [Version 1] doi: 10.12688/f1000research.191181.1 (PMC13694928; doi:10.12688/f1000research.191181.1)
Supplement: Peer review history

# Reviewer report: Reviewer response for version 1

**DOI:** 10.5256/f1000research.211009.r529517

**Published:** 2026-11-02

**Authors:** Elnetthra Folly Eldy

**Affiliations:** 1 Universiti Malaysia Sabah, Kota Kinabalu, Sabah, Malaysia

1. Rational and objectives of the study are partially clear. 

 2. The inclusion criteria cover inquiry-based and/or collaborative learning, while the title emphasize an integrated inquiry-collaborative model. Authors should resolve this inconsistency. 

 3. Explain why discovery learning, project-based learning, and problem-based learning are included within the inquiry-collaborative scope. 

 4. Explain why the PICo framework was selected, given that the review also compares educational outcomes and laboratory modalities. 

 5. The search strategy is described as having high sensitivity but low precision. provide sufficient information to support this assessment. 

 6. There is inconsistency between the search restrictions, which included conference papers, and the final eligibility criteria, which excluded them. This should explain more explicitly. 

 7. Authors should consider whether important studies using terms such as 5E learning cycle, POGIL, or PhET could have been missed. 

 8. Explained how the indpendence of screening decisions was maintained when co-authors reviewed existing decisions and exclusion notes. 

 9. The manual audit recovered 22 included reports that were missed during the initial screening. This raises concerns about the realibitlity of the original screening strategy. 

 10. The exclusion of 23 reports because full texts could not be retrieved may introduce availability bias. Explain the implications for the representativeness of the final evidence based. 

 11. Authors should explain more clearly how the appraisal results influenced the interpretation of study findings. 

 12. Clarify whether the 23 reports that could not be retrieved were potentially eligible or confirmed eligible. Eligibility cannot be fully established without examining their full texts. 

 13. Report how many record were recovered during the second screening and explain their characteristics. 

 14. Explain the implications of the relatively high representation of Indonesian studies for the international applicability of the findings. 

 15. Explain why the MMAT design categories differ substantially from the research design categories reported in Figure 3. 

 16. The statement that measurements is the strongest methodological area requires careful interpretation because appropriate instruments do not necessarily indicate valid causal inference. 

 17. Section 3.3 particularly relevant to the main objectives. 

 18. Clarify the criteria used to assign each study to one dominant pedagogical model and technological category. 

 19. Explains how studies using multiple teaching models or technologies were classified. 

 20. The finding that online nine reports explicitly desinged collaboration is important and should be emphasized. 

 21. Provide clear criteria for distinguishing genuine collaborative learning from ordinary group work. 

 22. Consider presenting a separate summary of the studies that genuinely integrated inquiry-based and collaborative learning. 

 23. This would strenghen the manuscript's contribution to understanding inquiry-collaborative pedagogical models. 

 24. The classification of learning outcomes into several compentency categories is useful. 

 25. Authors should avoid interpreting nonsignificant differences as evidence that virtual and physical laboratories are equivalent. 

 26. The claim that blended laboratories outperform single-mode laboratories should be supported with the number of direct comparison studies and the direction of their findings. 

 27. Where available, present effect sizes alongside statistical significance to support the interpretation of outcomes. 

 28. The results in Section 3.4 currently emphasize conceptual understanding more strongly than collaborative learning, despite the manuscript's central focus. 

 29. Explain whether the lack of collaborative outcome measurements prevents the review from answering its main research questions fully. 

 30. For Section 3.5, avoid describing all influencing factors as moderators unless supported by appropriate statistical analyses. 

 31. Consider separating pedagogical factors, learner-related factors, and institutional or technological constraints. 

 31. For discussion, several interpretations repeat the reseults rather than critically explaining them. 

 - This manuscript identifies collaboration as its most significant research gap. This finding deserves deeper theoretical discussion. 

 - Explain why collaborative learning is frequently incorporated into instructional activities but rarely assessed as an outcome. 

 - Discuss how future research might evaluate collaboration separately from conceptual learning. 

 Overall, the manuscript has the potential to contribute to the literature on physics education and technology-supported learning. However, substantial revisions are needed to strengthen the methodological rigor, establish the originality of the review, and ensure that the conclusions are adequately supported by the evidence.

Are the rationale for, and objectives of, the Systematic Review clearly stated?

Partly

Is the statistical analysis and its interpretation appropriate?

Yes

If this is a Living Systematic Review, is the ‘living’ method appropriate and is the search schedule clearly defined and justified? (‘Living Systematic Review’ or a variation of this term should be included in the title.)

Not applicable

Are sufficient details of the methods and analysis provided to allow replication by others?

Partly

Are the conclusions drawn adequately supported by the results presented in the review?

Partly

Reviewer Expertise:

Educational technology, Physics Education

I confirm that I have read this submission and believe that I have an appropriate level of expertise to confirm that it is of an acceptable scientific standard, however I have significant reservations, as outlined above.

# Reviewer report: Reviewer response for version 1

**DOI:** 10.5256/f1000research.211009.r529521

**Published:** 2026-10-23

**Authors:** Lovy Herayanti

**Affiliations:** 1 Universitas Pendidikan Mandalika, Kota Mataram, Indonesia

The manuscript is adequate and suitable for publication, subject to a few minor revisions.

1. The title highlights *“Inquiry-Collaborative Models,”* yet the findings indicate that collaboration was explicitly designed in only 15.3% of reports and measured in only 8.5%. Please clarify at the end of the Introduction that this review aims to critically examine the empirical gap between claims of collaboration and its actual implementation in practice.
2. In the *Limitations* section on page 11, the following sentence is duplicated: *“Finally, 23 eligible reports (20.9% of those sought) could not be obtained; two measured the collaborative process.”* Please remove one of the duplicate sentences.
3. Please clarify in the manuscript whether all MMAT scores were manually verified and cross-checked by the human research team to ensure the transparency and reliability of the appraisal process.

Are the rationale for, and objectives of, the Systematic Review clearly stated?

Yes

Is the statistical analysis and its interpretation appropriate?

Yes

If this is a Living Systematic Review, is the ‘living’ method appropriate and is the search schedule clearly defined and justified? (‘Living Systematic Review’ or a variation of this term should be included in the title.)

Not applicable

Are sufficient details of the methods and analysis provided to allow replication by others?

Yes

Are the conclusions drawn adequately supported by the results presented in the review?

Yes

Reviewer Expertise:

Science education; blended learning; learning management system; thinking skills

I confirm that I have read this submission and believe that I have an appropriate level of expertise to confirm that it is of an acceptable scientific standard.

# Reviewer report: Reviewer response for version 1

**DOI:** 10.5256/f1000research.211009.r529520

**Published:** 2026-10-23

**Authors:** Gunawan Gunawan

**Affiliations:** 1 University of Mataram, Mataram, Indonesia

This Systematic Literature Review (SLR) manuscript demonstrates a solid PRISMA reporting structure and provides detailed synthesis data.

 Please review the consistency of the main text. In the Abstract and Methods sections, the study period is stated as 2017–2026, whereas the opening sentence of Section 5 (Conclusions) refers to 2018–2026. Please ensure that the study period is reported consistently throughout the manuscript.

 This manuscript does not fall within the category of a Living Systematic Review.

Are the rationale for, and objectives of, the Systematic Review clearly stated?

Yes

Is the statistical analysis and its interpretation appropriate?

Yes

If this is a Living Systematic Review, is the ‘living’ method appropriate and is the search schedule clearly defined and justified? (‘Living Systematic Review’ or a variation of this term should be included in the title.)

Not applicable

Are sufficient details of the methods and analysis provided to allow replication by others?

Yes

Are the conclusions drawn adequately supported by the results presented in the review?

Yes

Reviewer Expertise:

Physics education; virtual laboratory; technology education

I confirm that I have read this submission and believe that I have an appropriate level of expertise to confirm that it is of an acceptable scientific standard.
